# Flexible dispersion engineering using polymer patterning in nanophotonic waveguides

**DOI:** 10.1038/s41598-023-40372-6

**Published:** 2023-08-14

**Authors:** Pei-Hsun Wang, Shang-Pu Wang, Nien-Lin Hou, Zong-Ren Yang, Wei-Hao Huang, Tien-Hsiang Lee

**Affiliations:** Department of Optics and Photonics, National Central University, Taoyuan City, 320317 Taiwan

**Keywords:** Nanophotonics and plasmonics, Microresonators, Silicon photonics

## Abstract

We demonstrate the engineering of waveguide dispersion by lithographically patterning the polymer cladding on silicon nitride waveguide resonators. Both normal and anomalous dispersion, ranging from − 462 to 409 ps/nm/km, can be achieved for the same waveguide dimension within an integrated photonic chip. In the meantime, this simple process shows no impact on the waveguide loss and the quality factor of the waveguide resonators, offering flexibility in tailoring designable dispersion for a universal photonic platform. In addition, by adjusting the coverage ratio of cladding, relatively low dispersion (≈ − 130 ps/nm/km) is also demonstrated in the same waveguide resonator, yielding the potentials for zero-dispersive waveguide resonators by a proper coverage ratio of the polymer cladding.

The rapid development of silicon photonics provides integrated solutions of miniature, portable, and mass-productive platforms of chip-based optical functionalities. By confining light propagation in the waveguide core layer, integrated waveguides pave the way for optical applications in optical communication^1^, optical sensing^2^, detection^3^, signal processing^4^, and nonlinear optics^5^. Today, with the aid of the CMOS-compatible fabrication process, large-scale integration enables the realization of hybrid, compact photonics. Among all of the on-chip devices, the group velocity dispersion (GVD) of optical waveguides is an essential factor in many applications, such as nonlinear optics, ultrafast optics, and optical communications. Traditionally, the waveguide dispersion can be adjusted with different waveguide geometries and this method has been widely applied in planar waveguides^6–8^. For instance, normal dispersion can be tuned to anomalous dispersion with a thicker waveguide on the silicon nitride platform^7,8^. Although the waveguide geometry can effectively compensate for the material dispersion and offer designable GVD, it loses the flexibility for the waveguide design and trades off the waveguide loss and available waveguide modes. To alleviate this restriction, several works are aimed at engineering the dispersion by cladding thin films onto waveguides with atomic layer deposition (ALD)^9^, sputtering^10^, or thermal evaporation^11^. However, this method globally clads the waveguides and defines the dispersion within the entire photonic chip, which is not suitable for engineering the dispersion of individual devices. To locally define the designable dispersion, several alternatives have been proposed to pattern waveguides with specific structures, such as patterning oxide cladding with hydrofluoric acid (HF) wet etching^12^, post-trimmings^13^, or designing cladding-modulated Bragg gratings^14^. Nevertheless, these approaches complicate the fabrication process or the layout design. Additional etching/trimming processes also restrict the available materials for the waveguide core. For example, even with a comparable quality (Q) factor of lithium niobate (LN) waveguides after patterning oxide cladding^12^, HF wet etching may easily damage Si_3_N_4_^15^ or GaAs waveguides^16^. A recent work proposed the capability to flexibly adjust waveguide dispersion using SU-8 polymer as the cladding layer^17^. The patternable feature of the polymer cladding enables the waveguide dispersion to be engineered in a reconstructable manner.

In this work, we demonstrate waveguide engineering with the polymer cladding layer on Si_3_N_4_ waveguide resonators. In comparison to the previous findings, our works result in several new achievements. First, with the SU-8 polymer patterning, the waveguide dispersion can be interpolated to a designable value. We experimentally show the waveguide dispersion with different coverage ratios at 0%, 25%, 50%, and 100%, revealing that the dispersion values are consistent with those obtained through linear interpolation. Second, for a fully cladding resonator with lithography patterning, similar intrinsic quality (Q) factors can be achieved in comparing to that without polymer cladding, showing negligible loss from the cladding layer. In the meantime, the multi-cycle polymer stripping process is demonstrated to validate the reconstructability of dispersion engineering while maintaining negligible impact on waveguide loss. Third, we successfully realize waveguide dispersion both in normal and anomalous regimes with the same waveguide dimension. Utilizing a thick Si_3_N_4_ waveguide, the dispersion can be tailed from anomalous (409 ps/nm/km) to normal (− 462 ps/nm/km) regime by increasing the coverage ratio of polymer cladding. Last, based on the above demonstrations, a relatively low dispersion ≈ − 130 ps/nm/km can be achieved by reconstructing the cladding with a 50% coverage ratio. It provides the potential to yield a zero-dispersive waveguide with a proper coverage ratio. These findings are significant for establishing photonic waveguides with varying dispersion values for different applications on the same integrated photonic chip.

## Device fabrication, simulation, and optical measurement

### Device fabrication

**Figure 1:**
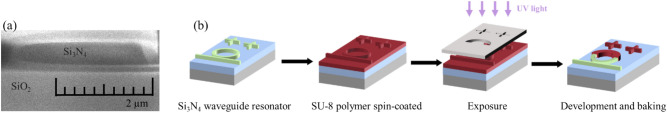
(**a**) The SEM image of the fabricated Si_3_N_4_ waveguides with a cross-section 500 nm × 3 μm. (**b**) The fabrication process of the polymer-cladding layer.

First, a 4 μm thick silicon oxide (SiO_2_) layer was thermally grown on silicon wafers in a diffusion furnace. Then, we deposited 500 nm/700 nm Si_3_N_4_ thin films on wafers with low-pressure chemical vapor deposition (LPCVD), respectively. For the 500-nm thick Si_3_N_4_ waveguides, e-beam lithography (EBL) was used to pattern the waveguide resonators with a negative-tone resist (ma-N 2405). After that, the devices were dry-etched in a high-density plasma etching tool. For the 700-nm thick waveguides, i-line (365 nm) stepper was used to pattern the waveguide with a positive-tone resist (Sumitomo PFI38). The devices were then dry-etched in a reactive ion etching tool. A focused ion beam (FIB) image of the fabricated Si_3_N_4_ waveguide with a cross-section 500 nm × 3 μm is shown in Fig. 1a.

**Figure 2:**
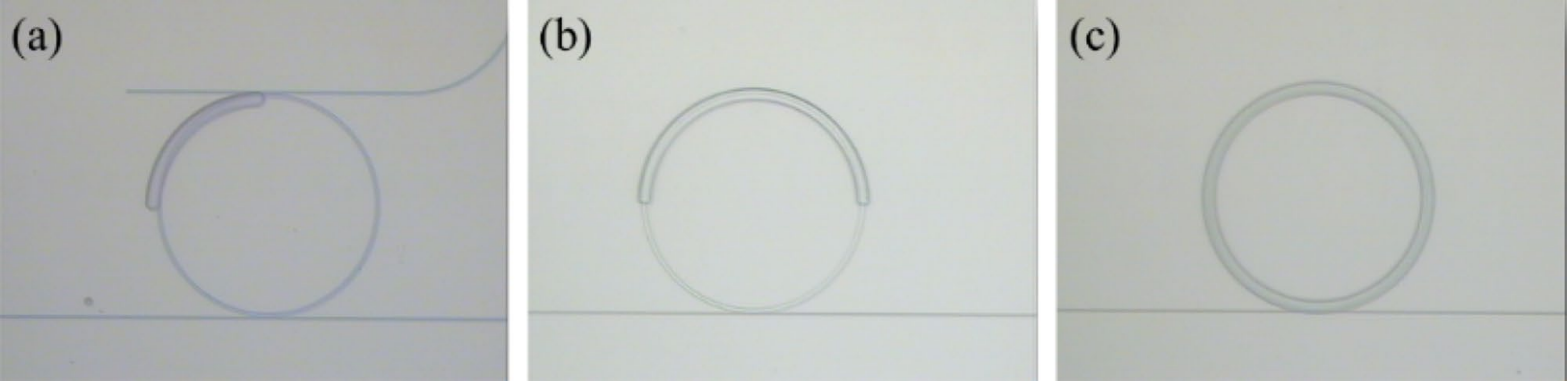
Images of the fabricated devices with (**a**) 25%, (**b**) 50%, and (**c**) 100% coverage ratios of the polymer-cladding layer.

As for the polymer-cladding layer, the fabrication process is shown in Fig. 1b. SU-8 polymer (GM1040) was first spin-coated on the air-cladded resonators with a thickness ≈ 1.7 μm. Then, this polymer-cladding layer was patterned by the conventional mask aligner and exposure system. The fabricated devices with a polymer-cladding layer are shown in Fig. 2. The width of the polymer-cladding layer is 10 μm. To have the SU-8 polymer as part of the final device, a 135 °C hard bake is performed for 2 h to avoid the surface cracks and increase the cross-linking density^18^. This process will also help to improve the Q factor of the polymer-cladded resonators.

### Waveguide dispersion simulation

**Figure 3:**
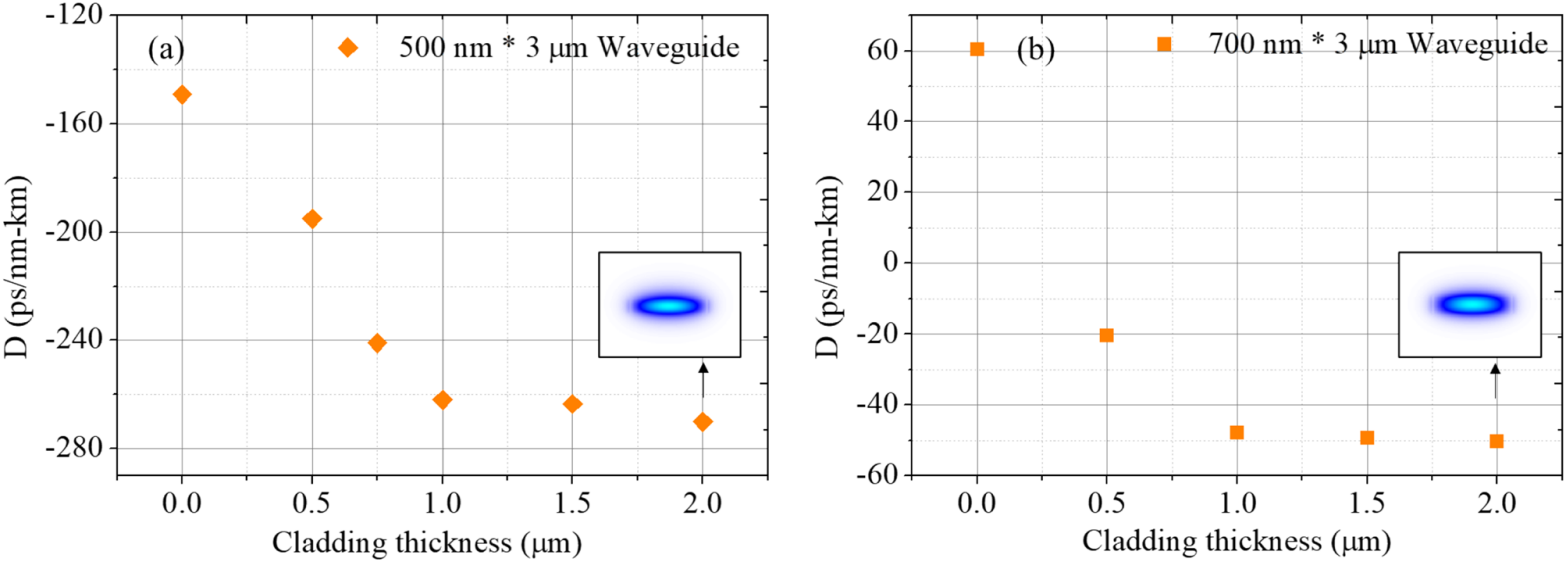
Simulated waveguide dispersion with varied SU-8 thicknesses. (Insets) The mode profiles with a 2 µm SU-8 polymer layer.

To verify the capability of dispersion engineering by the polymer cladding, we first explore the dependence of the SU-8 thickness on the dispersion. Figures 3 show the simulated waveguide dispersion with varied SU-8 thicknesses by incorporating both the dispersion curves of SU-8 polymer^19^ and Si_3_N_4_ thin films^20^. For a Si_3_N_4_ waveguide with a cross-section 500 nm × 3 μm, the waveguide dispersion can be adjusted from − 149 ps/nm-km (without SU-8 polymer) to − 269 ps/nm-km (with a 2 µm SU-8 polymer layer); when considering a waveguide with a cross-section 700 nm × 3 μm, the dispersion can be fine-tuned from 60 ps/nm-km (without SU-8 polymer) to − 50 ps/nm-km (with a 2 µm SU-8 polymer layer). The dispersion tuning shows a strong dependence on the varied thickness when the cladding thickness is less than 1 µm. For thicknesses ranging from 1 to 2 µm, the impact becomes less significant as there is no further overlap observed between the waveguide mode and the relatively thicker SU-8 polymer layer. The corresponding mode profiles with a 2 µm SU-8 polymer layer are shown in the insets. To adjust the waveguide dispersion by patterning the coverage ratio, a thicker SU-8 layer is preferred to avoid undesired variations caused by the fabrication process of the polymer spinning. This explains the employment of a 1.7 μm spin-coated SU-8 layer in this study.

### Optical measurement setup

**Figure 4:**
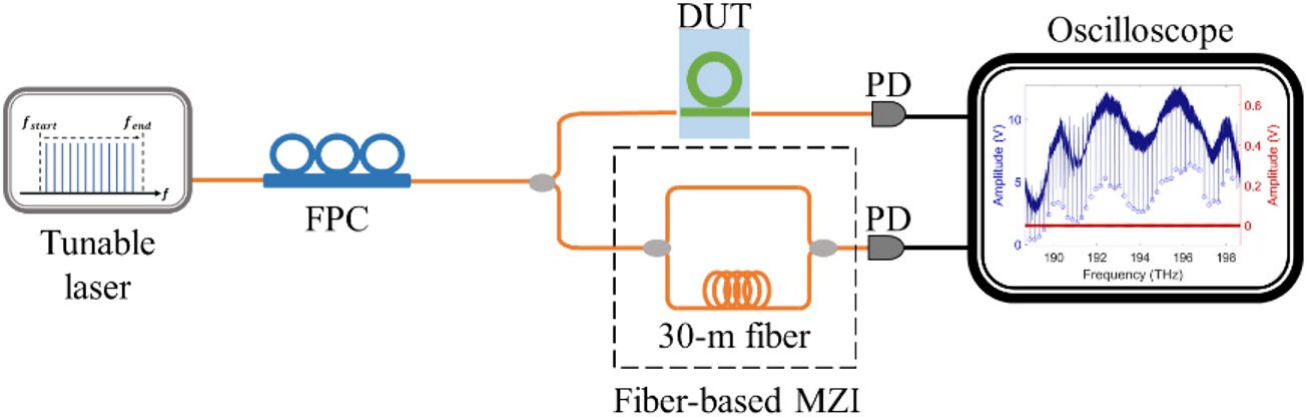
Schematics of the measurement setup.

The measurement setup is shown in Fig. 4. A tunable laser with a tuning range ≈ 80 nm around 1550 nm was used as the light source for transmission characterization. By coupling light into and out of the Si_3_N_4_ waveguides with a pair of lens fibers, the output signal is then recorded with an oscilloscope. Meanwhile, we use the fiber-based interferometry with 30-m optical path difference to generate interference fringes, providing the calibration markers of the transmission spectrum. The period of the generated interference fringes is around 6.89 MHz, offering better spectral resolution than that from the tunable laser system. Thus, this interference spectrum not only calibrates the instability of laser sweeping but also benefits the Q-factor characterization of an ultra-high Q device^21^.

## Dispersion engineering with polymer claddings

### Dispersion interpolation in normal dispersion via different coverage ratios of polymer cladding

For waveguide resonators, the dispersion can be evaluated by the frequency differences between the adjacent free-spectral-range (FSR) of the cavity. The relation is expressed by the following equation^17,22^:

1 $${\varvec{D}}=\frac{{\varvec{c}}}{2{\varvec{\pi}}{{\varvec{\lambda}}}^{2}{\varvec{R}}\cdot {{\varvec{F}}{\varvec{S}}{\varvec{R}}}^{3}}\frac{{\varvec{d}}{\varvec{F}}{\varvec{S}}{\varvec{R}}}{{\varvec{d}}{\varvec{m}}}$$

where *R* is the radius of the resonator, *c* is the speed of light, *m* is the azimuthal mode number, *λ* is the optical wavelength, and $\frac{dFSR}{dm}$ characterizes the difference between the adjacent FSRs.

**Figure 5:**
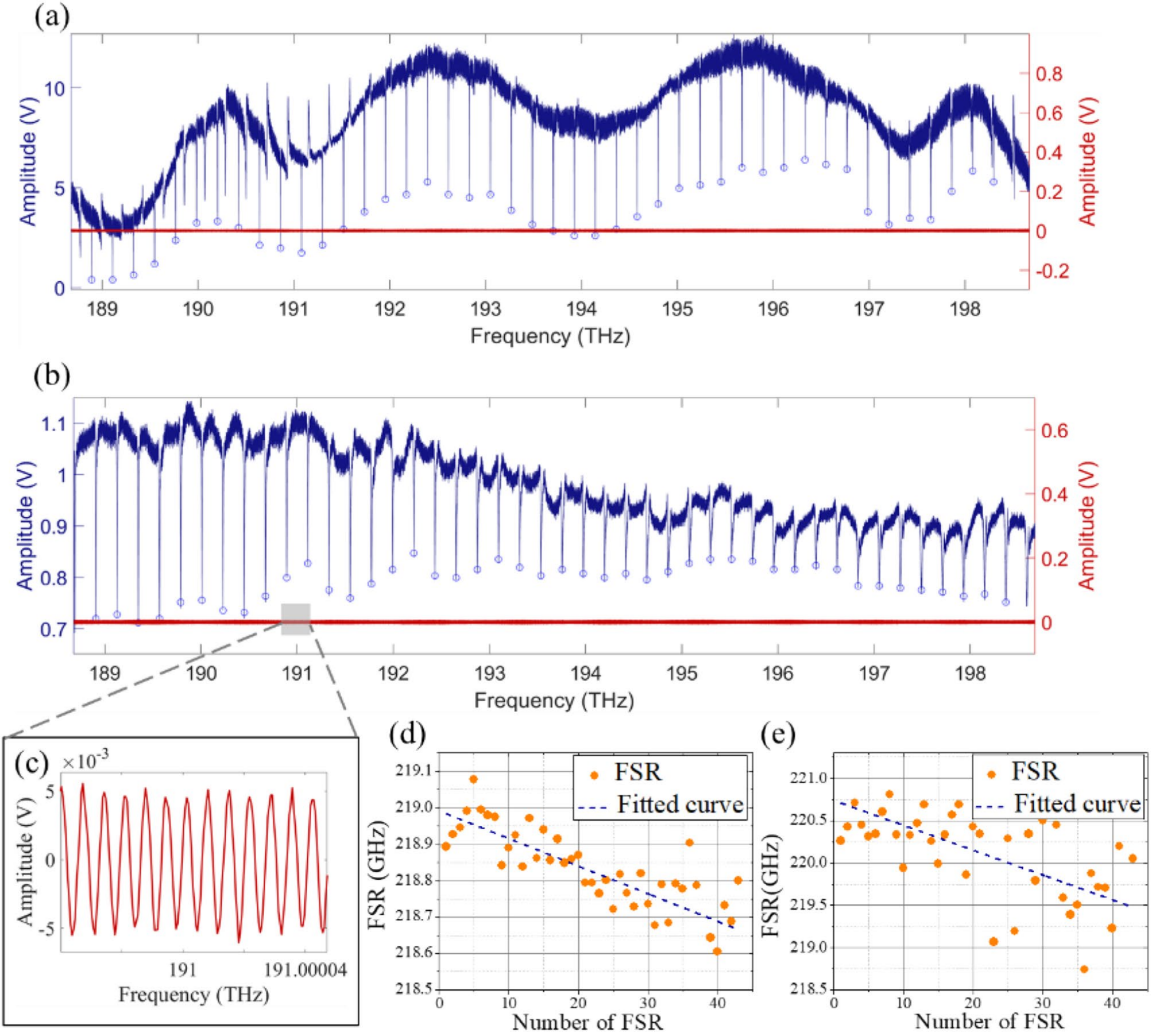
Transmission spectra of waveguide resonators with (**a**) air-cladding and (**b**) polymer-cladding. (**c**) The zoom-in spectrum of the interference fringes. The evolution of subsequent FSRs for (**d**) air-cladded and (**e**) polymer-cladded devices.

In this section, we study Si_3_N_4_ waveguide resonators with a cross-section 500 nm × 3 μm and a radius 100 μm. Traditionally, this waveguide exhibits normal waveguide dispersion^7,8^. Figures 5a,b show the measurement results of air-cladded and polymer-cladded waveguide resonators, respectively, while Fig. 5c is the zoom-in interference spectrum of the fiber-based interferometry. At around 1550 nm wavelength, the Lorentzian fitting yields the intrinsic quality factor (Q_i_) ≈ 1.6 × 10^5^ (air-cladding) and ≈ 1.1 × 10^4^ (polymer-cladding without patterning). The corresponding evolution of subsequent FSRs of the fundamental TE mode is shown in Fig. 5d,e, starting with the FSR located at the lowest sweeping frequency. For the air-cladded waveguide, the difference of the linear-fitted FSR is 7.3 MHz/FSR. As for the one with polymer cladding, the fitted results of FSR difference shift to 14.7 MHz/FSR, showing stronger normal dispersion.

**Figure 6:**
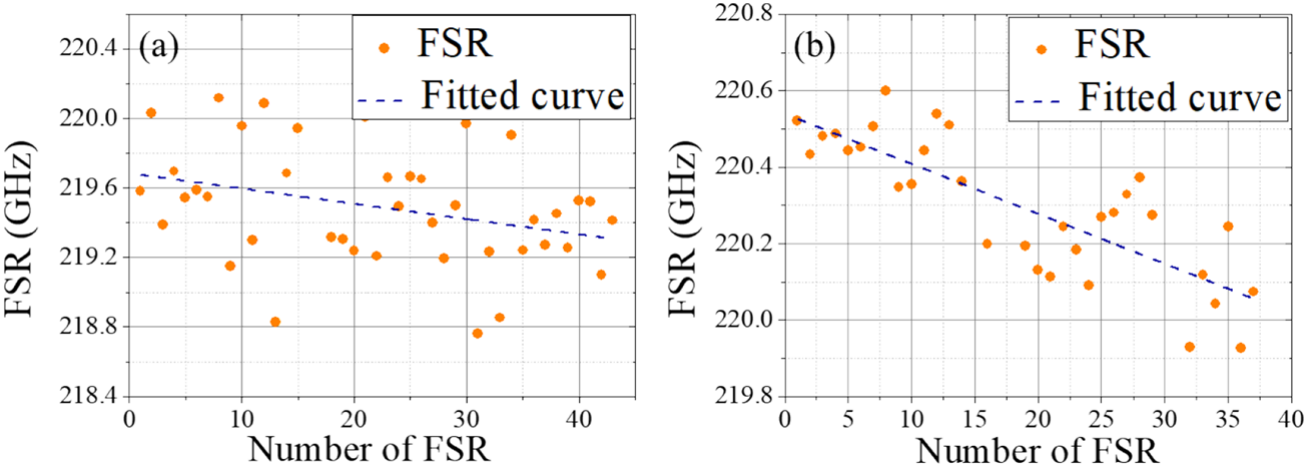
The evolution of subsequent FSRs for devices of (**a**) 25% and (**b**) 50% coverage ratios.

**Figure 7:**
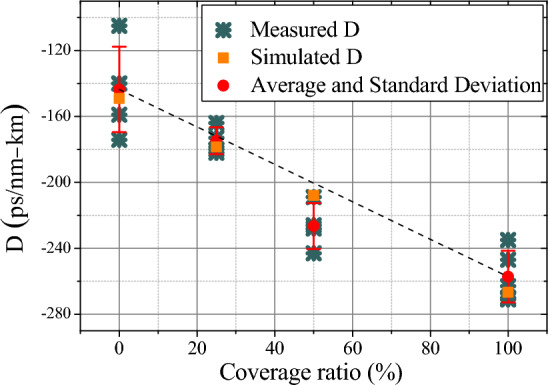
The measured dispersion of the waveguide resonators with a cross-section 500 nm × 3 μm. 0%, 25%, 50%, and 100% coverage ratios are demonstrated for the polymer-cladding.

The waveguide dispersion is then interpolated by patterning different coverage ratios of the polymer cladding layer. 25% and 50% coverage ratios are demonstrated by conventional ultraviolet (UV) contact lithography. The measurement results are shown in Fig. 6. With a 25% coverage ratio, the intrinsic quality factor slightly decreases to Q_i_ ≈ 4.8 × 10^4^ and the fitted FSR difference is 8.9 MHz/FSR. As for the 50% coverage ratio, the intrinsic quality factor Q_i_ ≈ 7.0 × 10^4^ with the fitted FSR difference around 13.0 MHz/FSR. In order to investigate the waveguide dispersion with polymer patterning, we show the measured dispersion of the resonators with 0%, 25%, 50%, and 100% of coverage ratios within the same chip in Fig. 7. For the air-cladded device with 0% coverage ratio, the measured dispersion parameter is − 143 ± 26 ps/nm-km, which agrees well with the simulated value − 149 ps/nm-km from the finite element method. As for the 100% polymer-cladded resonator, the dispersion is − 257 ± 16 ps/nm-km with the simulated value − 267 ps/nm-km. This polymer cladding tailors the dispersion deeper in the normal dispersion regime. When the resonator was partially polymer-patterned with 25% and 50% coverage ratios, the measured dispersion parameter is then interpolated in between, showing − 175 ± 8 ps/nm-km with the simulated value − 179 ps/nm-km and − 226 ± 14 ps/nm-km with the simulated value − 208 ps/nm-km, respectively, which qualitatively agrees with the simulated waveguide dispersion. This identification suggests that the dispersion parameter can be flexibly linear-interpolated within a varied dispersion range by patterning the coverage ratio of the polymer cladding layer.

### Flexible dispersion engineering for both anomalous and normal dispersion

**Figure 8:**
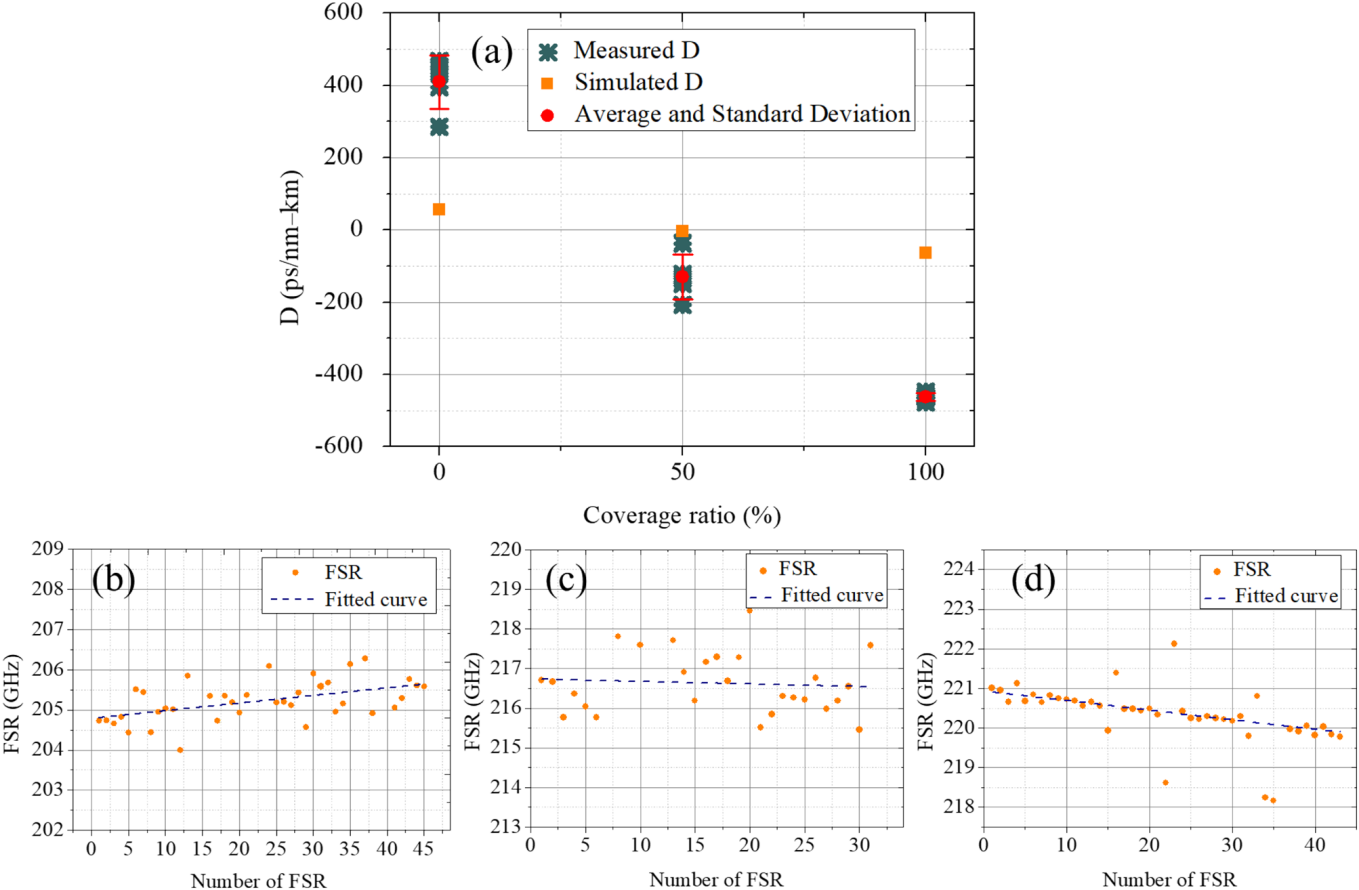
(**a**) The measured dispersion of the waveguide resonators with a cross-section 700 nm × 3 μm. 0%, 50%, and 100% coverage ratios are demonstrated for the polymer cladding. The corresponding evolution of subsequent FSRs for devices with (**b**) 0%, (**c**) 50%, and (**d**) 100% coverage ratios.

Next, we demonstrate the engineering for waveguide dispersion from anomalous to normal dispersion within the same device. To realize anomalous dispersion, waveguide resonators with a cross-section 700 nm × 3 μm and radius 100 μm are used in the air-cladded geometry. By patterning the polymer cladding on resonators, the measurement results of the fitted dispersion are shown in Fig. 8a, and the corresponding evolution of subsequent FSRs are shown in Fig. 8b–d for 0%, 50%, and 100% coverage ratios, respectively. As increasing the patterning ratio, the measured waveguide dispersion is then tuned from anomalous (409 ps/nm/km) with air-cladding to normal dispersion (− 462 ps/nm/km) with fully polymer-cladding; while for the device with 50% coverage ratio, the dispersion is interpolated at ≈ − 130 ps/nm/km. This large tuning range of dispersion provides the potential to realize a designable waveguide dispersion for different optical functionalities without the need for a specific waveguide dimension or geometry. Besides, by reconstructing the pattering ratio of the polymer cladding, nearly-zero dispersion could be achieved with a proper coverage ratio. This method provides flexibility to engineer waveguide dispersion for different applications.

## Discussion

**Figure 9:**
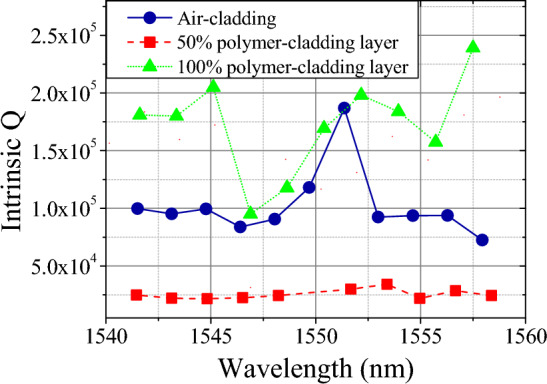
The distribution of intrinsic Q for air-cladded (blue), 50% polymer-cladded (red), and 100% polymer-cladded (green) resonators.

In this section, we further discuss the effect on the waveguide loss and the corresponding Q factors of the waveguide resonators with the polymer cladding. Figure 9 shows the fitted Q factors from the measurement results of 700 nm × 3 μm resonators. For the air-cladded device, the average intrinsic Q is ≈ 1.0 × 10^5^; while for the 50% and 100% polymer-cladded devices, the average Q_i_ is 2.5 × 10^4^ and 1.7 × 10^5^, respectively. The slight increasement in the Q factor of the 100% cladded device can be attributed to the better light confinement similar to that of an oxide-cladded waveguide^23^. In contrast to the observed degradation of Q factors from 10^5^ to 10^4^ in the spin-coated device without polymer patterning, as mentioned in^17^, the demonstrated device is processed by lithographically patterning the polymer around the resonator area. Here, the intrinsic Q factor of the device with 100% coverage remains consistently greater than 10^5^. In the case of the device with 50% cladding coverage, the degradation of Q factor can be attributed to the discontinuity of the polymer cladding. This leads to scattering loss in the propagating mode due to the resulting discontinuity of the traveling wave. To address this issue, a polymer taper can be introduced between the regimes with and without the polymer for mode matching and low-loss conversion, similar to the tapered waveguides previously used in fiber-to-waveguide interconnection^24^. Moreover, since the fabricated waveguide resonators support multi-mode propagation, the mode coupling between different mode families results in the abrupt change of Q factors at specific wavelengths^25^.

**Figure 10:**
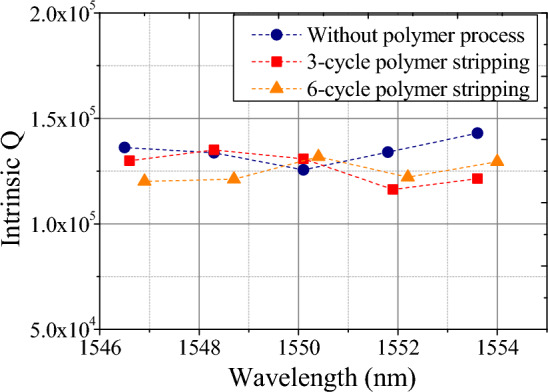
The distribution of intrinsic Q for waveguide resonators without the polymer process (blue), with 3-cycle polymer stripping (red), and with 6-cycle polymer stripping (orange).

Next, we study the susceptibility of the waveguide Q factors with the polymer stripping process. In^17^, the one-time stripping process demonstrates no impact on the intrinsic Q, highlighting the reconstructability of dispersion engineering. Here, we further investigate the influence of a multi-cycle SU-8 stripping process on waveguide loss. Figure 10 shows the measured intrinsic Q of waveguide resonators without the polymer process, with 3-cycle polymer stripping, and with 6-cycle polymer stripping. The stripping process is done by a photoresist remover (PUST-A01). The intrinsic Qs of the waveguides subjected to multi-cycle polymer stripping remain consistently comparable, indicating a high level of confidence in the reconstructable polymer patterning.

**Figure 11:**
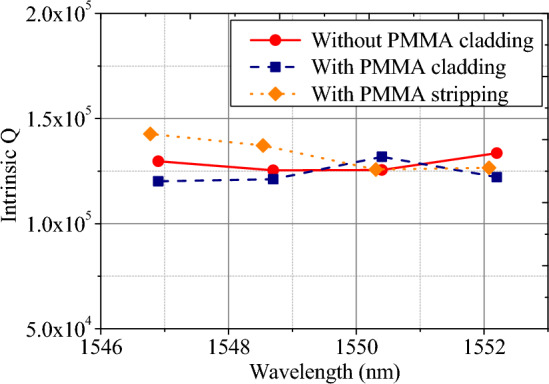
The distribution of intrinsic Q for waveguide resonators without the PMMA cladding (red), with the PMMA cladding (blue), and with PMMA stripping (orange).

To further emphasize the idea of dispersion engineering by the polymer cladding, another well-known, versatile lithographic resist, polymethyl methacrylate (PMMA) is also used here to verify the impact on the waveguide Q. This polymer is traditionally used in EBL and its rigid behaviors show stability to both acid and alkaline media^26^. By spin-coating ≈ 200 nm PMMA on the Si_3_N_4_ waveguide resonators, we show the measured intrinsic Q without and with PMMA cladding in Fig. 11. Again, due to the low absorption of PMMA at telecommunication bands, the intrinsic Qs are in a similar order after cladding the PMMA polymer. Also, after the PMMA stripping process, the waveguide resonators exhibit similar intrinsic Qs, verifying the reconstructability of polymer cladding.

Last, we compare different schemes of dispersion engineering for Si_3_N_4_ integrated waveguides reported previously and summarize in Table 1. Traditionally, by designing the waveguide geometries, the waveguide dispersion can be well tailored in both normal and anomalous dispersion regimes. However, this scheme loses the flexibility to optimize the waveguide geometries for better waveguide coupling or loss minimization. Also, it cannot offer different dispersion for each individual device on the same chip unless a complicated fabrication, such as trimming or etching, is applied^12,13^. As for the dispersion engineering by cladding with ALD deposition or sputtering, either the tuning range is limited (< 100 ps/nm/km), or precise control of the cladding thickness is needed. Meanwhile, the dispersion is globally defined within the entire chip when the fabrication process is typically time-consuming. In addition, for all the previous approaches, the dispersion is not reconstructable. With the polymer cladding, our previous work demonstrates the potential for dispersion reconstructability with patternable polymer cladding^17^. Here, we realize the dispersion tailoring in both normal and anomalous dispersion through conventional lithography patterning. Also, for the first time, the waveguide dispersion of the same waveguide geometry can be flexibly interpolated in between that with and without polymer cladding.

**Table 1:** Comparison of dispersion engineering for Si_3_N_4_ waveguides.

Scheme	Demonstrated dispersion (ps/nm/km)	Q	Dispersion regime	Reconstructability
Waveguide geometry				
^7^	447 to − 674	N/A	Normal/Anomalous	No
^8^	≈ 80 to 0	N/A	Anomalous	No
^27^	≈ 118 to 0	N/A	Anomalous	No
^28^	− 70	N/A	Normal	No
ALD/Sputtering cladding				
^9^	52 to − 27	10^6^	Normal/Anomalous	No
^10^	− 219 to − 827	10^6^	Normal	No
^29^	46 to − 213	10^5^	Normal/Anomalous	No
Patternable polymer				
^17^	− 143 to − 257	1.6 × 10^5^ (10^4^ with polymer cladding)	Normal	Yes
This work	**409 to **− **462**	1.0 × 10^5^ (**1.7 × 10**^**5**^ with polymer cladding)	**Normal/Anomalous**	**Yes**

Significant values are in bold.

## Summary

In conclusion, we demonstrated the dispersion engineering of Si_3_N_4_ waveguide resonators by patterning polymer cladding with conventional UV-contact lithography. The waveguide dispersion can be tuned from − 462 to 409 ps/nm/km and interpolated both in normal and anomalous dispersion regimes. Moreover, we showed the potential to obtain relatively low dispersion by patterning a proper coverage ratio of the polymer cladding. Due to the flexibility of polymer patterning, different waveguide dispersion can be locally assigned to the distinct devices of an integrated photonic chip without deliberately altering the individual waveguide geometry. This proposed scheme opens up the possibility of compact integrated optical devices for both linear and nonlinear optical functionalities.

## Data Availability

The data used in this study are available from the corresponding author upon reasonable request.
